# Scale-Free Dynamics of the Mouse Wakefulness and Sleep Electroencephalogram Quantified Using Wavelet-Leaders

**DOI:** 10.3390/clockssleep1010006

**Published:** 2018-10-20

**Authors:** Jean-Marc Lina, Emma Kate O’Callaghan, Valérie Mongrain

**Affiliations:** 1 Research Centre and Center for Advanced Research in Sleep Medicine, Hôpital du Sacré-Coeur de Montréal (CIUSSS-NIM), 5400 Gouin West blvd., Montreal, QC H4J 1C5, Canada; 2 Centre de Recherches Mathématiques, Université de Montréal, C.P. 6128, succ. Centre-Ville, Montreal, QC H3C 3J7, Canada; 3 École de Technologie Supérieure, 1100 rue Notre-Dame Ouest, Montreal, QC H3C 1K3, Canada; 4 Department of Neuroscience, Université de Montréal, C.P. 6128, succ. Centre-Ville, Montreal, QC H3C 3J7, Canada

**Keywords:** sleep regulation, vigilance state identification, multifractal formalism, time-of-day effect, Neuroligin-1, mice

## Abstract

Scale-free analysis of brain activity reveals a complexity of synchronous neuronal firing which is different from that assessed using classic rhythmic quantifications such as spectral analysis of the electroencephalogram (EEG). In humans, scale-free activity of the EEG depends on the behavioral state and reflects cognitive processes. We aimed to verify if fractal patterns of the mouse EEG also show variations with behavioral states and topography, and to identify molecular determinants of brain scale-free activity using the ‘multifractal formalism’ (Wavelet-Leaders). We found that scale-free activity was more anti-persistent (i.e., more different between time scales) during wakefulness, less anti-persistent (i.e., less different between time scales) during non-rapid eye movement sleep, and generally intermediate during rapid eye movement sleep. The scale-invariance of the frontal/motor cerebral cortex was generally more anti-persistent than that of the posterior cortex, and scale-invariance during wakefulness was strongly modulated by time of day and the absence of the synaptic protein Neuroligin-1. Our results expose that the complexity of the scale-free pattern of organized neuronal firing depends on behavioral state in mice, and that patterns expressed during wakefulness are modulated by one synaptic component.

## 1. Introduction

The electroencephalogram (EEG) represents a rich and evolutionary-conserved signal that indexes how brain activity is coordinated to perform information processing and memory storage [1]. The analysis of scale-free brain activity has revealed an unexpected dynamic complexity of synchronous neuronal firing of the human brain, different from that assessed using classical rhythmic quantifications such as spectral analysis [2,3]. This is because it captures the full range of brain oscillatory activities as well as a specific relationship between processes with different time scales. Indeed, contrary to rhythmic oscillations, scale-free activity (often modeled as fractal processes) reflects a spectral organization that does not depend on a specific temporal scale as the ‘scale-free’ property enforces a regular spectral decay without favoring any frequency. Scale-free activity has been shown to be a property of neuronal networks [4], and can be quantified using different methods such as nested-frequency analysis or detrended fluctuation analysis [2,5]. However, recent models considering the multifractal nature (i.e., the dynamics/variability) of scale-free activity seems to be particularly robust in estimating the complexity of a given signal including the EEG [6,7,8,9,10].

Importantly, behavioral states and cognitive processes were shown to modulate the so-called fractal patterns of the human and non-human primate EEG [2,7,9,10,11,12]. In fact, the fractal complexity (or anti-persistence) of the EEG, defined as the irregularity of the signals between various temporal scales, was shown to be lower during non-rapid eye movement (NREM) sleep than during rapid eye movement (REM) sleep or wakefulness in humans [7,9,11]. Moreover, scale-free activity was shown to express a lower complexity (i.e., a higher persistence) in anterior brain regions during different sleep states [11]. The study of topographic patterns of scale-free activity of the sleep EEG and of the underlying molecular mechanisms will further the knowledge on the established role for sleep in memory consolidation and in physiological health [13,14]. Cortical scale-free dynamics were already shown to be linked to learning and information processing [2], which involve the coordination of neuronal activity in multiple brain regions such as the hippocampus and neocortical areas [15,16]. In parallel, in addition to behavioral states, internal (circadian) time influences cognitive performance and memory [17], which should associate with optimal 24 h patterns of scale-free activity in relevant brain regions given the association between scale-free activity and cognitive performance [2]. Thus, understanding the daily dynamics and topography of scale-free activity of the wakefulness and sleep EEG, as well as the underlying molecular mechanisms, will contribute to unraveling the integrated manner by which the brain processes and stores information.

Neuroligins (NLGNs) are postsynaptic adhesion molecules interacting with presynaptic partner Neurexins (NRXNs) and regulating synaptic maturation and activity [18]. NLGN1 is predominantly localized at excitatory synapses and controls the functioning of ionotropic glutamate receptors [19,20]. Glutamate transmission was proposed to contribute to scale-free activity of cerebral cortex assemblies [2]. NLGN1, in particular, has been implicated in spatial learning and associative fear memory [21,22]. In addition, NLGNs have been linked to sleep regulation both in the fruit fly and mouse [23,24]. Indeed, we observed that *Nlgn1* knockout (KO) mice express an impaired capacity to sustain wakefulness [24]. Furthermore, *Nlgn1* KO mice showed lower theta and alpha synchrony during wakefulness, and enhanced delta wave synchrony during NREM sleep especially after prolonged wakefulness [24,25]. Therefore, NLGN1 not only participates in mechanisms underlying alternation of behavioral states but also in mechanisms mediating synchronized neuronal activity during wakefulness and sleep.

Here, we aimed to verify four hypotheses using a recent approach to analyzing scale-invariance with the Wavelet-Leaders formalism [26,27] applied on cortical EEG recording in wild-type and *Nlgn1* KO mice: (1) Scale-free properties of the mouse EEG differ between behavioral states (wakefulness, NREM sleep, REM sleep) in a similar manner to what is observed in humans; (2) also similar to humans, behavioral state-dependent EEG scale-free activity in the mouse is modulated by topography because neuronal assemblies show topographic specificity; (3) behavioral state-dependent EEG scale-free activity in the mouse varies with time-of-day since the quality of wakefulness and sleep varies as a function of daytime; and (4) the absence of NLGN1 will impact on EEG scale-free activity in a behavioral state-dependent manner. This was performed on a sub-sample of EEGs previously recorded in *Nlgn1* KO mice and littermates [24]. Our findings support these hypotheses and represent an important step forward in the identification of molecular determinants of brain scale-free activity.

## 2. Results

### 2.1. Scale-Invariance Measured Using the Multifractal Formalism

**Figure 1:**
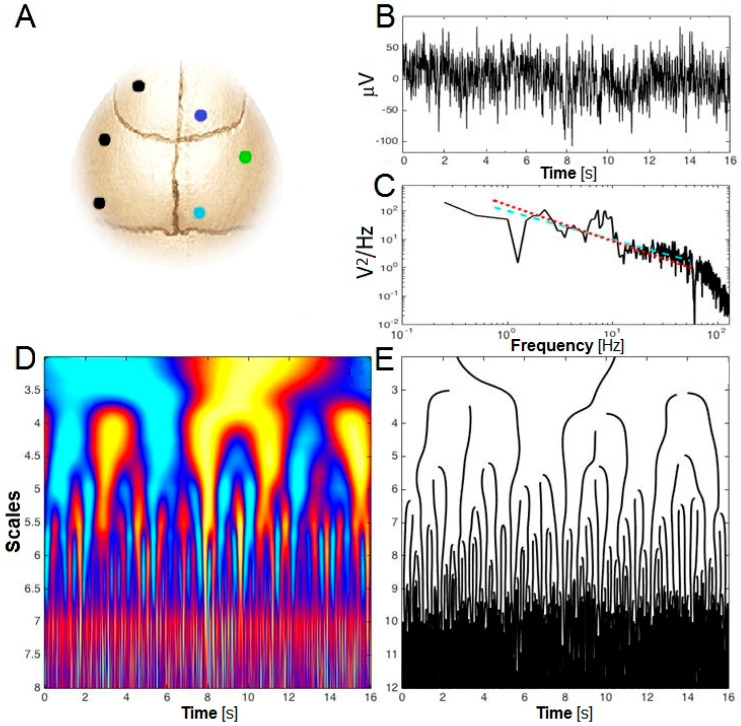
Electrode positioning and quantification of scale-free dynamics. (**A**) Schematic view of the mouse skull indicating the position of the anterior (dark blue dot) and posterior (pale blue dot) EEG electrodes and of the reference EEG electrode (green dot) over the right hemisphere. Black dots indicate the position of anchor screws; (**B**) Example of a typical wakefulness time series lasting 16 s; (**C**) Fourier spectrum depicted on a log-log scale for the time series presented in (**B**). The slope of the linear regression (dashed blue line) is −1.009 (i.e., *H* = 0.5) whereas the multifractal formalism gives $H_{m}=0.134$ (i.e., a slope equal to −1.27; dashed red line); (**D**) Continuous wavelet-based time-frequency representation of the time-series presented in (**B**); (**E**) Lines of local maxima (spectral crests) of the time-frequency plan (**D**). Along each line, the wavelet coefficients behave as a local -in time- Fourier analysis. The original multifractal formalism consisted in analyzing the scaling property of those maxima along the lines. The recent Wavelet-Leaders formalism revisits the formalism in the discrete wavelet framework and provides a robust estimation of the dominant scaling exponent *H_m_* (i.e., the slope of the dashed red line in (**C**)).

Fractals were originally used to describe geometric shapes or time series showing reproducible irregular patterns at all (or a wide range of) scales. Here, we characterized the scaling properties and the variations across time of the irregularities of the EEG signal at two electrode locations over the right hemisphere of the mouse cerebral cortex (Figure 1A). The spontaneous EEG time series (Figure 1B) does not exhibit a characteristic scale, but rather exhibits a power spectrum with a power-law decay of the form $1/f^{\gamma }$ parametrized with a characteristic scaling exponent γ (Figure 1C). A generalization of such processes has been proposed where this exponent is no longer unique (e.g., monofractal) but varies with time and spreads over a specific range (i.e., multifractal). We used the Wavelet-Leaders formalism to perform multifractal analysis (see Methods). The original continuous wavelet approach mimics a spectral analysis localized in time (Figure 1D) [28]. Along each maxima line in the time-scale (or time-frequency) plane (Figure 1E), the wavelet coefficients describe local fluctuations at a particular time and scale, and behave as $f^{-\gamma }$ in case of a self-similar process endowed with a unique exponent. Here, we applied the more recent Wavelet-Leaders formalism [8], because of its robustness in the estimation of the local Hurst exponent (*H*) from which the scaling exponent is computed by γ=2H+1 [6]. Since H may be not unique along the time series, the multifractal formalism derives a so-called ‘singularity spectrum’ over all possible values of *H*. We will denote by $H_{m}$ the *H* that is most prevalent in the analyzed time series and by D the dispersion of *H* around $H_{m}$. The greater the absolute value of *D*, the more multifractal (and complex) the time series is. Note that in Figure 1C, D is almost zero (i.e., monofractal) and the estimate of *H_m_* would lead to a scaling exponent *γ* that would be similar to a *γ* provided by a naive linear regression of the log-log spectrum.

### 2.2. Scale-Invariance is More Anti-Persistent During Wakefulness

**Figure 2:**
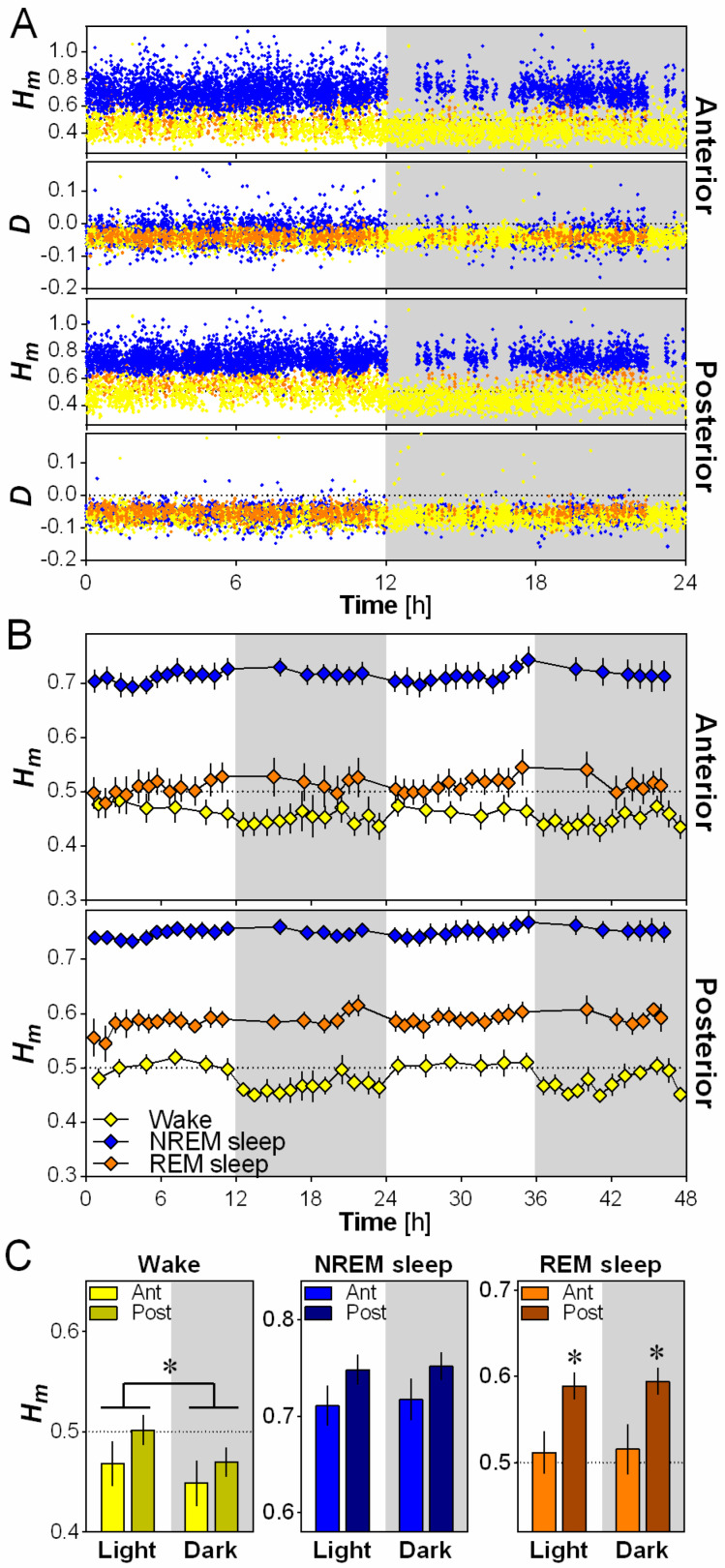
Scale-invariance of the mouse cerebral cortex according to behavioral state, electrode position and time of day. (**A**) Scaling exponent *H_m_* and dispersion index *D* for anterior and posterior electrodes for one wild-type mouse (all 4-s epochs of all behavioral states during 24-h baseline). Wakefulness epochs are indicated by yellow dots, NREM sleep by blue dots and REM sleep by orange dots; (**B**) Mean 48-h time course of *H_m_* computed separately for wakefulness, NREM sleep and REM sleep, and for the two electrodes in wild-type mice (*n* = 6). *H_m_* was averaged across the two days for each interval to compute statistical comparisons between behavioral states. ANOVAs reveal a significant effect of behavioral state for both the anterior (F_2,10_ = 49.7, *p* < 0.001) and posterior (F_2,10_ = 64.2, *p* < 0.001) electrodes; (**C**) *H_m_* calculated for the two electrodes separately during the 12-h light and 12-h dark periods for the three behavioral states in wild-type mice (*n* = 6). ANOVAs reveal a significant effect of light/dark for wakefulness (F_1,5_ = 31.1, *p* < 0.01), and a significant effect of electrode for REM sleep (F_1,5_ = 15.3, *p* = 0.01). *: *p* < 0.05 between indicated points.

To investigate the difference in scale invariance between behavioral states, *H_m_* and *D* were calculated for the anterior and posterior electrodes for every 4-s epoch over 24 h, separately for wakefulness, NREM sleep and REM sleep (Figure 2A). A higher *H_m_* is indicative of more persistence (less complexity/variability between time scales) in scale-free activity and a lower *H_m_* is indicative of more anti-persistence (higher complexity/variability between time scales), with *H_m_* = 0.5 being generally considered as the value that distinguishes persistence from anti-persistence [6]. Representative data from one mouse indicates that *H_m_* generally varied between 0.25 and 1, with very little overlap between wakefulness and NREM sleep values (Figure 2A). The sign of the dispersion index *D* validated the applicability of the multifractal formalism given that a negative value of *D* is required by the multifractal model (see Materials and Methods). In general, for the present analysis, *D* values were lower than 0 for both the anterior and posterior electrodes, and completely overlapped between wakefulness, NREM sleep and REM sleep (Figure 2A).

For both the anterior and the posterior electrodes, *H_m_* was consistently higher during NREM sleep than during wakefulness and REM sleep in wild-type mice (generally NREM sleep 0.70–0.75, wakefulness 0.45–0.50, REM sleep 0.50–0.60; Figure 2B), indicating more persistence (i.e., less variability across time scales) in scale-free activity during NREM sleep compared to the other states. *H_m_* was also significantly higher in REM sleep than wakefulness for the posterior electrode only. Thus, wakefulness showed higher anti-persistence of scale-free activity (or higher complexity) than other behavioral states in the mouse cerebral cortex.

### 2.3. Scale-Invariance Varies with Electrode Location and Daytime

We then evaluated the effect of cerebral cortex topography and that of the light and dark periods on *H_m_* using 12-h averages (Figure 2C). A significant effect of electrode location was only observed for REM sleep, for which *H_m_* was higher in the posterior than in the anterior location. This indicates a higher anti-persistence (complexity) of scale-free activity in the anterior cerebral cortex region than in the posterior during REM sleep. On the other hand, a significant effect of daytime was only found for wakefulness, showing a higher *H_m_* during the light than during the dark period. This indicates that, for a given electrode location, the mouse wakefulness EEG is not always expressing the same scale-free pattern, and that it shows an elevated anti-persistence (i.e., more variability across time scales) during the active (dark) period compared to the rest (light) period.

### 2.4. NLGN1 Absence Increases Scale-Free Anti-Persistence During Wake

**Figure 3:**
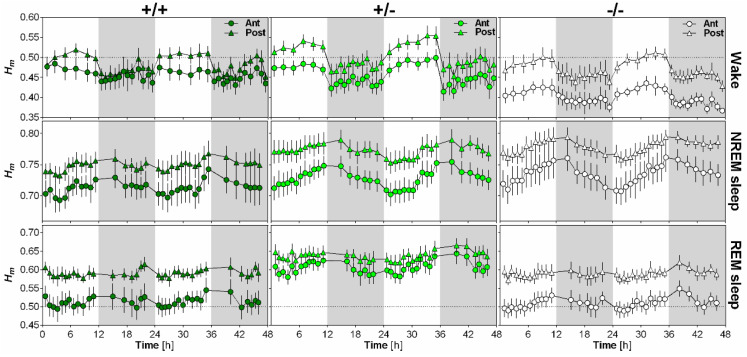
Impact of NLGN1 absence on scale-free patterns of the mouse EEG. 48-h time course of the scaling exponent *H_m_* is represented for wakefulness (first row), NREM sleep (second row) and REM sleep (third row), separately for the anterior electrode (circles) and the posterior electrode (triangles) in wild-type (+/+, *n* = 6; first column), heterozygous (+/−, *n* = 8; second column) and *Nlgn1* KO (−/−, *n* = 8; last column) mice. For wakefulness, ANOVA reveals a significant effect of time (F_17,323_ = 18.6, *p* < 0.001), and significant interactions between genotype and electrode (F_2,19_ = 3.6, *p* < 0.05) and between electrode and time (F_17,323_ = 6.3, *p* < 0.001). For NREM sleep, a significant time effect (F_17,323_ = 18.0, *p* < 0.001), and a significant interaction between electrode and time (F_17,323_ = 5.0, *p* < 0.001) were found. For REM sleep, ANOVA reveals significant effects of genotype (F_2,19_ = 6.2, *p* < 0.01) and time (F_17,323_ = 6.1, *p* < 0.001), and a significant interaction between electrode and time (F_17,323_ = 5.1, *p* < 0.001).

The impact of NLGN1 on the scaling exponent *H_m_* and its time course was analyzed separately for wakefulness, NREM sleep and REM sleep using EEG recordings of wild-type (+/+), heterozygous (+/−) and *Nlgn1* KO (−/−) mice (Figure 3). First, *H_m_* was clearly and significantly affected by time for all behavioral states, with values peaking during the light phase for wakefulness, at light-dark transition for NREM sleep and generally during the dark phase for REM sleep. In addition, these time courses differed according to electrode position. Secondly, scale-free activity during wakefulness was particularly affected by the absence of NLGN1, since KO mice showed lower *H_m_* than wild-type and heterozygous mice. However, this difference was only found for the anterior electrode. Moreover, this resulted in a higher difference in *H_m_* between the anterior and posterior electrodes in KO mice than in other genotypes. Interestingly, scale-free activity during REM sleep was also significantly affected by the mutation, but differences from wild-type mice were only observed in heterozygous mice. More precisely, *H_m_* was significantly higher in heterozygous mice than in wild-type and KO mice during REM sleep, and this difference was not significantly modulated by electrode or time. Overall, the total absence of NLGN1 boosted scale-free complexity in anterior cerebral cortex during wakefulness, while *Nlgn1* haploinsufficiency decreased scale-free complexity during REM sleep.

### 2.5. Opposite Relationship between Wake Scale-Invariance and NREM Sleep Delta Activity in Nlgn1 KO Mice

**Figure 4:**
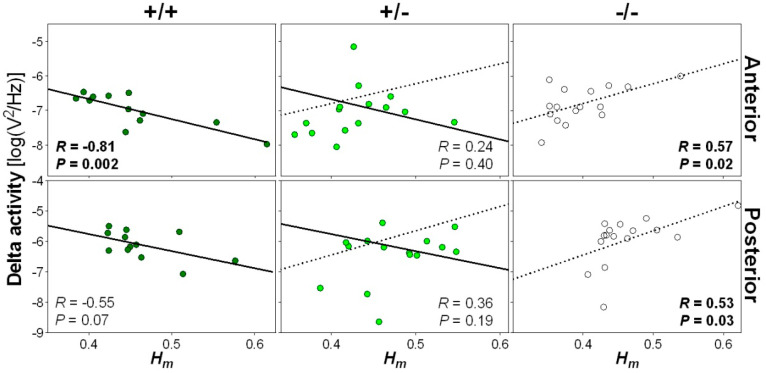
Relationship between wake scale-free activity and NREM sleep delta power. *H_m_* was computed for wakefulness epochs occurring during the 7th hour of the dark periods and delta activity (1–4 Hz EEG activity) was averaged for NREM sleep epochs over the 8th hour of the dark period for the two recording days, and these values were correlated separately for wild-type (+/+, left panels), heterozygous (+/−, middle panels) and *Nlgn1* KO (−/−, right panels) mice. Top and bottom rows show correlations for anterior and posterior electrodes, respectively. The solid line represents the regression line in wild-type mice, and the dotted line the regression line of KO mice. One datapoint missing for heterozygous mice since no NREM sleep was observed on the 8th hour of the first baseline. *R* and *P* values of significant correlations are highlighted in bold.

Given the substantial impact of the *Nlgn1* mutation on wakefulness scale-free activity, and our previous observation of amplified NREM sleep delta activity and oscillations in *Nlgn1* KO mice [24,25], we verified if wakefulness scale-free activity was associated with NREM sleep rhythmic slow activity (i.e., delta [1–4 Hz] activity). To ensure a temporal correspondence between these EEG variables, this was performed by averaging *H_m_* over an hour containing mostly wakefulness preceding an hour containing a considerable amount of NREM sleep (7th and 8th hours of the dark period, respectively; see [24]). In wild-type mice, a lower wake *H_m_* was significantly associated with higher subsequent NREM sleep delta activity at the anterior electrode location (Figure 4). A similar tendency was observed for the posterior electrode. Conversely, a lower wake *H_m_* was significantly associated with a lower delta power in *Nlgn1* KO mice. No significant association was found between wake *H_m_* and the following NREM sleep delta activity in heterozygous mice, but it is worth noting that some heterozygotes appear to align on the regression line of wild-type mice, while others on that of KO mice. These findings suggest that higher multifractal complexity of the EEG is linked to higher subsequent delta activity during NREM sleep in normal mice, but that the absence of NLGN1 reverses this relationship.

## 3. Discussion

We uncover here that scale-free dynamics of the rodent EEG varies as a function of behavioral state, electrode location, and time of day. Indeed, we found that the multifractal pattern during wakefulness in particular expresses more complexity between different time scales (i.e., more anti-persistence) than that during NREM sleep, and more complexity in the active (dark) period than during the rest (light) period. We also report that scale-invariance is generally less complex (i.e., more persistent) in the posterior cerebral cortex than the anterior cortex in mice. Importantly, we reveal that NLGN1, a post-synaptic component, specifically impacts scale-free activity during wakefulness providing evidence of a molecular pathway contributing to brain scale-free activity.

We found that brain scale-free dynamics are potently influenced by behavioral state in mice, showing more complexity (i.e., more anti-persistence) during wakefulness, less complexity (i.e., more persistence) during REM sleep, and minimal complexity during NREM sleep. This is similar to what has been observed for the human EEG [7,9,11]. Also similar to humans [7,10], the distinction between states seems robust enough (e.g., no overlap of *H_m_* between wakefulness and NREM sleep or between NREM and REM sleep) to be used for automatic identification of behavioral states, especially if combined with electromyographic (EMG) activity. In parallel, neuronal firing expresses scale-free activity [4], and it seems intuitive that more complexity (i.e., anti-persistence) would be more evident in wakefulness when animals are exposed to a variety of stimuli and show more complex behavior and information processing in comparison to sleep states when subjected to less sensory input. Recent literature described how the rapidly changing level of waking vigilance impacts cortical neuronal responsiveness and reaction to sensory stimulation [29], and this may be reflected in higher fractal complexity compared to sleep states. Our data thus supports that high complexity of scale-free activity that associates with a more disorganized/randomized network favors environmental responsiveness during wakefulness. On the other hand, the NREM sleep EEG, which is characterized by rhythmic synchronous activity of thalamo-cortical networks and reduced excitatory input to these regions [30,31], shows less complex activity compared to both wakefulness and REM sleep. The predominance of slow synchronous activity during NREM sleep increases the representation of slower scales, which may favor persistence between scales. Given that a highly turbulent network could potentially interfere with sleep, a higher persistence in network oscillations during deep sleep could serve to protect or preserve sleep.

In humans, state-dependent differences in antero-posterior EEG frequency distribution have been described [32], and lower complexity (i.e., higher persistence) of scale-free activity seemed to be found in anterior brain regions during sleep [11]. On the contrary, we demonstrated that in mice, scale-free dynamics show higher anti-persistence at the anterior electrode than at the posterior electrode for all behavioral states, indicating more complexity at the frontal location. This was especially evident for REM sleep during analysis of the light and dark phases. With our recording montage, the posterior electrode was recording mostly from the visual cortex, while the anterior electrode mostly from the motor cortex. This differs from the anterior brain area in humans that is mainly composed of a higher-order cognitive processing area, the prefrontal cortex, which could explain between-species differences. In parallel, the different organization of the mouse cerebral cortex in the motor versus visual region, for instance of inhibitory connections to excitatory neurons [33], may favor higher vs. lower fractal complexity, respectively. Besides, the posterior electrode is situated close to the hippocampus, which is thus expected to contribute to the scale-free pattern recorded at the level of the visual cortex. The hippocampus generates highly synchronized rhythms in a wide range of frequencies [34], which may contribute to less fractal complexity (i.e., more fractal persistence) at the posterior site. This may be particularly prominent during REM sleep, since hippocampal EEG synchronization increases during REM sleep [35]. Although fMRI and slice culture support scale-free properties of the hippocampus [2,36], further work will be required to explore the specific contribution of the hippocampus to scale-free EEG patterns.

The present findings also highlight that brain scale-free dynamics are greatly impacted by time of day. Indeed, scale-free activity during wakefulness is more complex (i.e., more anti-persistent) during the dark period in comparison to the light period (Figure 2C and Figure 3), and scale-free complexity during NREM and REM sleep generally decreases in the course of the light period and increases during the dark period (Figure 3). These observations could suggest either an influence of the light-dark cycle or of the endogenous circadian timing system. EEG activity was shown to be influenced by both light [37] and circadian processes [38]. The fact that the 24 h rhythm was equally observed for the anterior (motor) and posterior (visual) electrodes, and especially our observation that the 24 h rhythm is characterized by a gradual increase in the course of the light period, which was particularly evident for NREM sleep, could suggest a substantial contribution of the circadian system. Distinguishing between light-dark and circadian influences will require quantification of EEG scale-free dynamics under constant darkness conditions.

Our findings reveal a first molecular component affecting fractal complexity of the EEG. The absence of NLGN1 impacted scale-free dynamics of the wakefulness EEG, with KO mice showing increased complexity (i.e., anti-persistence) at the anterior electrode resulting in a larger anterior-posterior difference in complexity. This observation may indicate a decrease in network coordination between cortical regions in absence of NLGN1, and supplements our previous report of decreased rhythmic EEG activity in *Nlgn1* KO mice during wakefulness [24]. Studies have shown that NLGN1 deletion increased the excitability of neuronal networks, and this was attributed to less excitation of inhibitory neurons [39]. Increased excitability in the cerebral cortex may directly impair the capacity of cortical networks to generate persistent/more regular patterns of activity. This might be due to one of the two main functions of NLGN1 in excitatory synaptic transmission: the regulation of *N*-methyl-d-aspartate glutamate receptors (NMDAR) activity [19] and of presynaptic glutamate release in response to high neuronal activity [40]. Dysregulated glutamate release and NMDAR function in the absence of NLGN1 may disorganize neuronal firing, which could reflect in the fractal pattern as complexity (i.e., anti-persistence). These functions of NLGN1 could equally contribute to modifications in anterior-posterior connectivity leading to antero-posterior disconnection of fractal pattern during wakefulness.

Interestingly, heterozygous mice showed attenuated fractal complexity during REM sleep compared to both WTs and KOs. It is possible that a non-uniform distribution of NLGN1 between cells in REM sleep regulatory areas specifically in heterozygous mice could account for these results. Previously, a study showed that when differences exist in levels of NLGN1 between cells, high or low expression will result, respectively, in increased or decreased synapse number in the individual cell [41]. If these alterations are not observed across the neuronal population, as may be the case in heterozygotes, this could result in decreased synaptic density and potentially decrease fractal complexity (i.e., increase fractal persistence) during REM sleep. These *Nlgn1* haploinsufficiency-driven alterations may specifically affect REM sleep regulatory networks, in particular cholinergic networks, or factors regulating cholinergic transmission, since REM sleep is characterized by high activity of cholinergic neurons [42]. NLGN1 was indeed shown to contribute to cholinergic synapses in vitro [43]. In sum, data from both heterozygous and KO mice indicate impaired coordination of neuronal activity within and between cortical regions when NLGN1 is downregulated.

We also reveal that scale-free activity during wakefulness is associated with delta activity in subsequent NREM sleep. Under normal conditions, anti-persistent patterns, which may reflect high turbulence/fluctuations in neuronal activity, were followed by higher delta activity in NREM sleep. This might be anticipated if complexity (i.e., anti-persistence) proved particularly tiring for neuronal networks, given that delta activity has been used as the main index of homeostatic sleep need [44]. Moreover, this association strongly depended upon NLGN1. Indeed, we describe a reversal of the relationship between prior wakefulness scale-free complexity and subsequent NREM sleep delta activity in *Nlgn1* KO mice, with higher complexity (i.e., anti-persistence) of scale-free dynamics during wakefulness followed by lower delta activity. This contrasts our previous observation of higher delta activity under elevated sleep need in these mice [24,25], and may indicate that scale-free complexity specifically of extended wakefulness in *Nlgn1* KO mice is relatively low. Otherwise, it could represent an alternative to the brain’s restorative processes that normally follow periods of high neuronal activity, such as those in wake. It is interesting to note that such an inverse relationship between waking scale-free complexity and NREM sleep delta activity might also contribute to the reported difficulty to sustain wakefulness shown by these mice, as indexed by decreased time spent awake and shorter individual bouts of wakefulness [24].

In conclusion, besides its potential usefulness for the automatic identification of behavioral states, our findings reveal a greater complexity of organized network patterns of synchronous firing in wakefulness compared to sleep in the mouse, and indicate that this arrhythmic pattern is modulated by synaptic elements such as NLGN1. Our analysis of fractal activity provided information that was previously unattainable by spectral analysis of the EEG alone, in particular concerning effects of the *Nlgn1* mutation on the EEG [24,25]. Given the implication of NLGN1 in neuropsychiatric conditions, as revealed in autism spectrum disorder models [45], assessment of scale-free activity in psychiatric patients is warranted to understand the extent of brain network impairment in these pathological populations, and develop strategies to improve/restore scale-free dynamics.

## 4. Materials and Methods

### 4.1. Animals, Surgery and Protocol

B6;129-*Nlgn1^tm1Bros^*/J mice (i.e., adhesion protein NLGN1 absent due to deletion of coding exons 1 and 2 [46]) were purchased from Jackson Laboratories and bred on site. Mice were maintained with *ad libitum* food and water, 12 h/12 h light/dark cycle, and 23–25 °C ambient temperature, and studied between 10 and 16 weeks of age. Mice studied here (*n* = 8 *Nlgn1* KO [−/−], 8 heterozygous [+/−], and 6 wild-type [+/+]) represent a sub-sample of mice previously studied (*n* = 12 KO, 14 heterozygous and 10 wild-type in [24]) for which the referenced signal of the anterior and posterior electrodes was suitable for scale-free analysis. More precisely, animals for which the signal of the reference electrode (see below) was unusable (e.g., dysfunctional electrode, predominant artifacts) could not be included in the present study.

The electrode implantation surgery has thus been described elsewhere [24]. Briefly, gold-plated screws of 1.1 mm of diameter served as EEG electrodes and were screwed through the skull over the right cerebral hemisphere as illustrated in Figure 1A (anterior electrode: 1.2 mm lateral to midline, 1.5 mm anterior to bregma; posterior electrode: 1.2 mm lateral to midline, 1.0 mm anterior to lambda). A third screw implanted over the right hemisphere (2.6 mm lateral to midline, 0.7 mm posterior to bregma) served as a reference electrode for the anterior and posterior electrode signals. Two gold wires were inserted between neck muscles and served as EMG electrodes. The EEG and EMG were recorded for 48 h of baseline as detailed previously [24,47].

During the recovery from surgery and during the EEG/EMG recording, animals were individually housed in cages of 43 cm length by 22 cm width by 39 cm height, and had access to minimal environmental enrichment (i.e., sweet pellet for mice around the recording period) to minimize risk of damage to the head montage. Behavioral states (wakefulness, NREM sleep, and REM sleep) were visually assigned off line to 4-s epochs, and artifacts were simultaneously marked, using the EEG and EMG signals (animals were not video-recorded). The experiment was approved by the Ethical Committee for Animal Experimentation of the Hôpital du Sacré-Coeur de Montréal (CIUSSS-NIM).

### 4.2. Scale-Invariance and 1/f Power Spectrum

A scale-free signal is well illustrated by the self-similar random process x(t) endowed with the property:

$$x\left(at\right)\approx a^{H}\;x\left(t\right)$$

where a is an arbitrary scaling parameter. This equality should be understood in probability and states that a zoom-in (a>1) or a zoom-out (a<1) of the signal reproduces a signal similar to what the original process would have given at scale 1, up to a renormalization of the amplitude given by the Hurst exponent H>0. Thus, there is no characteristic scale. Due to its inherent non-stationarity, the analysis of a self-similar process is challenging. However, the sub-class of such processes with stationary increments can be defined. Indeed, given a time delay θ, stationary increments are defined by $x_{\theta }\left(t\right)=x\left(t+\theta \right)-x\left(t\right)$ with $x_{\theta }\left(t\right)$ $\approx x_{\theta }\left(0\right)$. This process reproduces the self-similar property, $x_{\theta }\left(at\right)\approx a^{H}\;x_{\theta }\left(t\right)$. The statistical moments of the increments are given by $E\left[{\left|x_{\theta }\right|}^{q}\right]\;\sim \;\theta^{qH}$ for q>−1. Furthermore, it was shown that autocovariance of the increments behave as follows (for large time delays τ) [48]:

$$\;E\left[x_{\theta }\left(t+\tau \right)\;x_{\theta }\left(t\right)\right]\;\sim \;H\;\gamma \;\theta^{2}\;\tau^{\gamma -1}$$

where γ=2H−1. This result has two important consequences. First, this process exhibits persistence or long-range dependency provided 0<γ<1, i.e., 1/2<H<1. Conversely, we obtain an anti-persistent process (with more complexity) when 0<H<1/2. *H* = 0.5 thus sets the threshold for persistence and anti-persistence of the fluctuations in the original time series [6]. Second, the power spectrum density of the increment process is of the form $\sim 1/f^{\gamma }$, whereas the power spectrum of the original signal x(t) is of the form $\sim 1/f^{\gamma +2}$, i.e., $\sim 1/f^{\upsilon }$ with υ=2H+1 (see example in Figure 1C).

### 4.3. Wavelet Analysis of Scale-Invariance 

The previous increments are a particular form of the fluctuations computed at a specific time scale defined by the parameter θ. A generalization of this analysis can be defined in terms of wavelet coefficients, either in the continuous time-frequency framework (Figure 1D), based on a ‘local Fourier-like analysis’ as illustrated with the maxima of Figure 1E [21], or the more recent approach in the multiresolution representation of the process [27]. The latter is defined through the discrete wavelet coefficients

$$d_{j}\left(k\right)=\;\int x\left(t\right)\;\Psi_{j,k}\left(t\right)dt\;\mathrm{with}\;\Psi_{j,k}\left(t\right)=2^{-j}\;\Psi \left(2^{-j}t-k\right)$$

(the increments $x_{\theta }\left(t\right)$ are obtained with the wavelet Ψ(t)=δ(t−1)−δ(t) and the scale $\theta =2^{j}$). The self-similarity property of the process x is now expressed on the associated wavelet coefficients,

$$d_{j}\left(k\right)\;\approx \;2^{jH}\;d_{0}\left(k\right)$$

where j indexes the scale $a=2^{j}$. The statistical moments (also called the structure function) $S_{q}\left(j\right)\overset{\mathrm{def}}{=}E\left[{\left|d_{j}\right|}^{q}\right]$ behave as the moments of an increment of a self-similar process, at scale $2^{j}$, that is, $S_{q}\left(j\right)\;\sim \;2^{jqH}$.

This framework, well established for an exact self-similar process (with a unique Hurst exponent H) can be generalized for processes that show not a unique but a range of exponents, h(t) reachable at various temporal samples. This generalization, usually called the multifractal formalism [28], consists in writing the structure function in the form $S_{q}\left(j\right)\;\sim \;2^{j\zeta \left(q\right)}$. The previous ‘monofractal’ case ζ(q)=qH is now generalized to a non-linear function that describes departure from the strict self-similar process. In the present work, we consider the quadratic expansion ζ(q) = $H_{m}\;q\;+\;1/2\;D\;q^{2}$ to generalize the modeling of scale-free dynamics, for which $H_{m}$ represents the Hurst exponent *H* that is most prevalent in the analyzed time series, and D thus quantifies the dispersion of Hurst exponents around $H_{m}$. A non-vanishing value of *D* indicates that the signal contains many different Hurst exponents. Therefore, a large absolute value of *D* indicates a more multifractal (and complex) time series. However, consistency of the model requires the convexity of ζ(q) and thus D≤0. Indeed, the quadratic term (with D<0) describes a departure from the strict self-similarity for which the power spectrum is strictly of the form $\sim 1/f^{2H+1}$. Thus, for D<0, the process is still endowed with scale-free property but it does not show a unique Hurst exponent that could have been estimated directly from the power spectrum.

### 4.4. The Wavelet Leaders Formalism

Scaling invariance in signals has been revisited in the Wavelet Leaders formalism [8,27]. This approach consists of redefining the previous multiresolution structure function using wavelet quantities that will reproduce at best the local Hurst exponent in the signal. As proposed previously [49], those quantities can be obtained from the previous wavelet coefficients by taking the largest value of the wavelet coefficients in the finer scales in the neighborhood. This redefinition of the multiresolution description of the signal allows estimating the structure function in a robust way for a wide range of values of q, including negative values, and thus leading to a reliable estimate of the parameters $H_{m}$ and D [27]. The numerical implementation of this formalism used in the present work is made available through the freely-accessible *Wavelet Leaders and Bootstrap to Multifractal Formalism* (WLBMF) MATLAB toolbox. A step-by-step script (MATLAB code) to be used with this toolbox to estimate *H_m_* and *D* for EEG data can be provided on request with a tutorial.

### 4.5. Multifractal Data Analysis and Spectral Analysis

The parameter *H_m_* was estimated for every artifact-free 4-s epoch, averaged for the 12-h light and 12-h dark periods and for intervals, for which an equal number of epochs contributed. This last calculation was performed to take into account wakefulness and sleep distribution: *H_m_* was averaged for 12 intervals during the light periods and 6 intervals during the dark periods for NREM sleep and REM sleep, and for 6 intervals during the light periods and 12 intervals during the dark periods for wakefulness. Finally, wakefulness *H_m_* was computed for the 7th hour of the dark periods to assess the relationship with delta power computed using Fourier transform during the following hour. The delta band was defined as 1 to 4 Hz, and spectral activity was averaged over all NREM sleep artifact-free epochs of the 8th hour of the dark periods.

### 4.6. Statistical Analyses

Comparisons of *H_m_* between behavioral states, electrodes, time of day and genotypes were performed on averages of the two recording days using two- or three-way repeated-measure analyses of variance (ANOVAs). Significant effects were decomposed, when appropriate, using Tukey tests or planned comparisons, and adjusted for repeated-measure using the Huynh-Feldt correction. Pearson correlations were used to assess relationship between *H_m_* and delta power using data from the two recording days. The significance threshold was set to 0.05, and data are reported as mean ± SEM.
